# Gravity-driven hydromagnetic flow of couple stress hybrid nanofluid with homogenous-heterogeneous reactions

**DOI:** 10.1038/s41598-021-97045-5

**Published:** 2021-09-01

**Authors:** Muhammad Waseem, Taza Gul, Imran Khan, Arshad Khan, Anwar Saeed, Ishtiaq Ali, Poom Kumam

**Affiliations:** 1 Department of Mathematics, City University of Science and Information Technology, Peshawar, KP Pakistan; 2 Department of Mathematics and Statistics, Bacha Khan University, Charsadda, Khyber Pakhtunkhwa 24420 Pakistan; 3 College of Aeronautical Engineering, National University of Sciences and Technology (NUST), Sector H-12, Islamabad, 44000 Pakistan; 4 Center of Excellence in Theoretical and Computational Science (TaCS-CoE), Faculty of Science, King Mongkut’s University of Technology Thonburi (KMUTT), 126 Pracha Uthit Rd., Bang Mod, Thung Khru, Bangkok, 10140 Thailand; 5 Department of Mathematics and Statistics, College of Science, King Faisal University, P. O. Box 400, Hafouf, 31982 Al-Ahsa Saudi Arabia; 6 Department of Medical Research, China Medical University Hospital, China Medical University, Taichung, 40402 Taiwan

**Keywords:** Applied mathematics, Fluid dynamics, Mathematics and computing, Physics

## Abstract

This investigation describes the hydromagnetic flow of gravity-driven couple stress hybrid nanofluid past a heated plate. The carbon nanotubes (CNTs) are used to characterize the hybrid nanofluid. The heated plate is placed vertically with an application of homogenous-heterogeneous reactions to the assumed flow system. The homogeneous reaction governs by isothermal cubic autocatalytic kinetics while the heterogeneous reaction governs by the first order kinetics. For current study the couple stress hybrid nanofluid is presumed to be conducted electrically with impact of non-uniform magnetic effects. An appropriate set of dimensionless quantities has employed to governing equations and then has solved by homotopy analysis method. The influence of emerging parameters encountered in this work has discussed in detail with the help of graphs. In this study it has examined that, flow of fluid reduces with upsurge in magnetic parameter and volumetric concentrations, whereas thermal and concentration characteristics augment with increase in volumetric concentrations. Moreover, growth in Prandtl number leads to a reduction in thermal characteristics and growth in Schmidt number result a reduction in concentration profile. The impact of various emerging parameters has also studied numerically upon physical quantities. It has established that, with augmentation in values of buoyancy parameter there is a growth in the values of skin friction. A comparison has also carried out between current and established results with a fine agreement in both results.

## Introduction

The fluids which are conducting electrically are named as magnetohydrodynamics (MHD) fluids such as liquid metals, electrolytes and plasma etc. The idea of MHD was first presented by a Swedish electrical engineer Alfven^1^. The MHD waves introduced by him are also known as Alfven waves. The basic idea in the rear of MHD is that the electric current is induced by magnetic effects through a conductive moving fluid. This type of fluid has numerous applications at industrial level such as reactor cooling, drug targeting etc. Many researchers and scientists have carried out a number of studies in the field of MHD. Alotaibi et al.^2^ examined numerically the effect of MHD Casson nanofluid flow upon a nonlinear convectively heated sheet influenced by viscous dissipative and suction-injection effects. Krishna and Chamkha^3^ have discussed the Hall effects and ion slip upon the MHD swirling flow for nanofluid. In this work the flow has considered past a porous and vertical plate using a constant heat source. Lund et al.^4^ examined the MHD flow for micropolar nanofluid past a shrinking and vertical sheet in the presence of buoyancy effects. Islam et al.^5^ inspected the impacts of thermally radiative Hall current upon MHD micropolar hybrid nanofluid flowing between two plates. In this work the base fluid is considered as blood with nanoparticles of grapheme oxide and copper. The readers can examined more about MHD fluid flow in Refs.^6–12^.

If the nano-sized particles of silver, copper, alumina etc. are suspended in some pure fluid is called nanofluid. It has established experimentally that, the coefficient of heat transmission for nanofluids is increased by combining the nanoparticles with some base fluids. For improving the thermal characteristics of base/pure fluid, the quantity of nanoparticles was first proposed by Choi^13^. Afterwards, many researchers have discussed different characteristics of nanfluids flow. Eid et al.^14^ discussed three dimensional Prandtl nanofluid flow with chemical reaction through some permeable materials. In this investigation the Brownian and thermophoresis effects have also considered to see the enhancement of heat transfer characteristics of the flow system. Al-Hossainny and Eid^15^ have examined the spinning reaction of mono and hybrid nanofluid upon an expanding surface. Hamid et al.^16^ have carried out a stability test for transfer of heat for MHD nanofluid using thermal radiations past a moving needle. In this article bvp4c technique has introduced for determination of approximate solution. Carbon nanotubes (CNTs) are normally prepared with the help of graphite. When hexagonal nano sized sheet are rolled up we obtain CNTs. It can be single or multi walled tubes also known as SWCNTs and MWCNTs. Their applications are categorized as enhancement of electrical and thermal conductivity and thermal stability etc. Khan et al.^17^ have discussed the entropy production for CNTs nanofluid flow amid two porous stretched revolving disks. In this work the lower and upper disks have assumed to be revolving with angular motion. Khan et al.^18^ have carried out an approximate solution for entropy production using peristaltic motion of single and multi-wall CNTs. It has established in this work that Brinkman number became a source for increasing the production of entropy for flow system. Javed et al.^19^ have analyzed the single and multi-walled nanotubes for heat transport by employing thermal radiation in a channel which was non-uniform. In this work viscosity has assumed to be a function of thermal characteristics and exact solution of modeled problem has established. Further investigation about use of CNTs in different flow can be examined in Refs.^3,20–26^.

Due to the extensive and useful applications of couple stress flows in numerous production processes, couple stress can be taken in non-Newtonian fluids, liquid crystals and animal blood, lubrication with polymer. Such applications comprise of rotary machinery, cooling in the fabrication of metal sheets in a bath. In fluid mechanics, the theory of couple stress has introduced in non-Newtonian fluids by Stokes^27^. The classical theory of viscous Newtonian fluids derived the fluid theory influenced by couple stress. The main concept of the couple stress theory is to show or analyzed the skin frictions amongst the particles. Khan et al.^28^ investigated the collective transmission of heat and mass through vertical channel. He obtained the solution of highly non-linear problems by HAM technique. Many investigations in literature^29–33^ are available about the couple stress fluids.

In this work, the MHD flow of hybrid nanofluid is examined above a vertically placed heated plate. The flow system is assumed under the influence of homogenous and heterogeneous reactions where the former reaction is governed by autocatalytic kinetics while the later one is governed by first order kinetics. Moreover, for current study the couple stress hybrid nanofluid is presumed to be conducted electrically with application of non-uniform magnetic field to flow problem. The modeled equations have transformed into dimensionless form by employing set of similar quantities and then have solved using HAM.

## Physical and mathematical description

**Figure 1:**
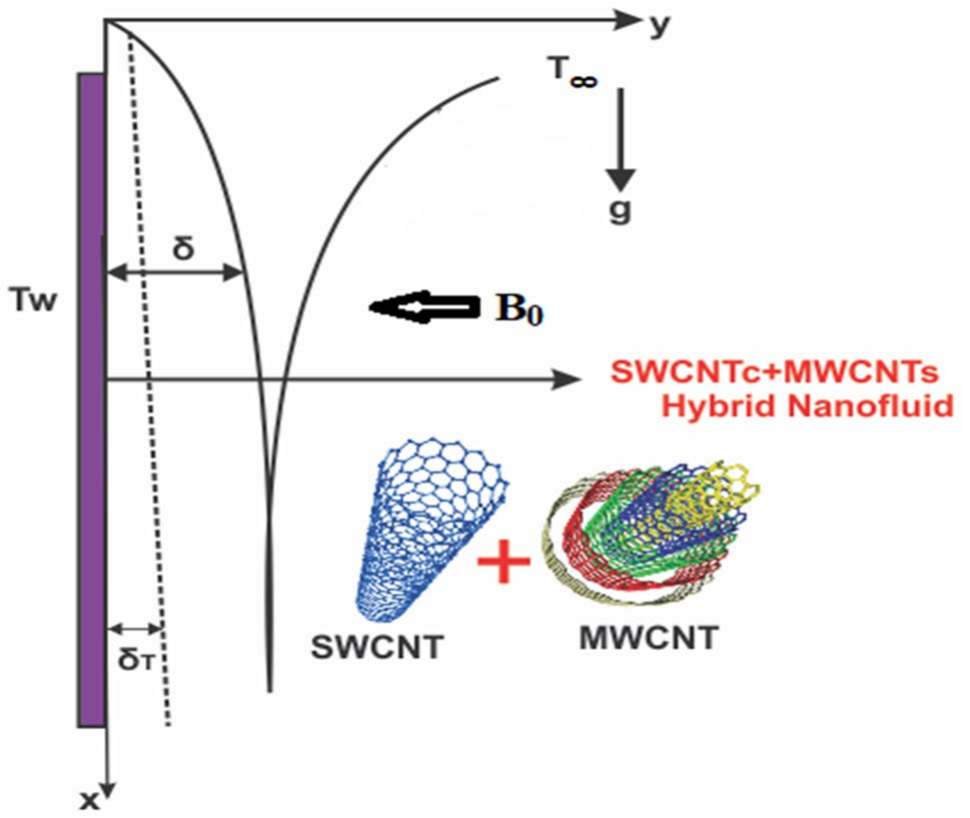
Graphical view of the flow problem.

Take a 2-D hybrid nanofluid flow under the influence of gravitational impact. The flow is taken above a vertically placed heated plate using homogenous/heterogeneous reactions (see Fig. 1). In the schematic diagram a rectangular coordinates system is selected with $x{\text{-}}axis$ in vertical downward direction of the plate whereas $y{\text{-}}axis$ is selected as in normal direction to the plate. Based on the model presented by Chaudhry and Markin^34^ the isothermal cubic autocatalytic reaction is systematically presented as

1 $$ A + 2B \to 3B,\,\,\;{\text{rate}} = k_{1} ab^{2} \; $$

On the catalyst surface heterogeneous reaction takes place and is given mathematically as

2 $$ A \to B,\;{\text{rate}} = k_{s} a $$

In Eqs. (1, 2) $A,\,B$ are the chemical species with $a,\,b$ as their concentrations whereas $k_{1} ,\,k_{s}$ are constants.

Keeping in view the stated supposition we have the flow equations as follows^35–37^

3 $$ \frac{\partial u}{{\partial x}} + \frac{\partial v}{{\partial y}} = 0, $$

4 $$ \rho_{hnf} \left( {u\frac{\partial u}{{\partial x}} + v\frac{\partial u}{{\partial y}}} \right) = \rho_{f} g + \mu_{hnf} \left( {\frac{{\partial^{2} u}}{{\partial y^{2} }}} \right) - \sigma_{hnf} B_{0}^{2} u - \eta \frac{{\partial^{4} u}}{{\partial y^{4} }} $$

5 $$ u\frac{\partial T}{{\partial x}} + v\frac{\partial T}{{\partial y}} = \alpha_{hnf} \frac{{\partial^{2} T}}{{\partial y^{2} }} + kcab^{2} \left( {\frac{\Delta Hh}{{\delta A}}} \right)\frac{1}{{\left( {\rho cp} \right)_{hnf} }} $$

6 $$ u\frac{\partial a}{{\partial x}} + v\frac{\partial a}{{\partial y}} = D1\frac{{\partial^{2} a}}{{\partial y^{2} }} - kcab^{2} , $$

7 $$ u\frac{\partial b}{{\partial x}} + v\frac{\partial b}{{\partial y}} = D2\frac{{\partial^{2} b}}{{\partial y^{2} }} + kcab^{2} , $$

8 $$ u\frac{\partial C}{{\partial x}} + v\frac{\partial C}{{\partial y}} = D_{hnf} \frac{{\partial^{2} C}}{{\partial y^{2} }}. $$

Here, the Oberbeck Boussinesq assumption is employed for balancing the relation amid $\rho_{hnf}$ and $\rho_{f}$ that is described as

9 $$_{{\rho hnf = \rho_{f} \left[ {1 - \beta \left( {T - T\infty } \right)} \right]}} $$

In Eq. (9) $\beta$ describes the thermal expansion coefficient while reference temperature is represented by $T_{\infty }$. It is to be noticed that $g$ (gravitational acceleration) as given in Eq. (4) is linked with downward velocity $U\left( x \right) = \sqrt 2 gx$ by pressure variation in *x*-direction as

10 $$ g = U\frac{\partial U}{{\partial x}} $$

The boundary conditions at $y = 0$ are stated as

11 $$ u = v = 0\; -{\text{k}}_{T} \frac{\partial T}{{\partial Y}} = k_{s} a\left( {\frac{\Delta Hh}{{\delta A}}} \right),\,\,C = Cw, \, D1\frac{\partial a}{{\partial y}} = - D2\frac{\partial b}{{\partial y}} = ksa; $$

whereas at y → ∞ these conditions are

12 $$ U = U\left( x \right),\;T \to T_{\infty } ,\;C \to C_{\infty} ,\;a \to a_{\infty } ,\;b \to 0 $$

Assume the stream function as $\psi$ with flow components as

13 $$ u = \frac{\partial \psi }{{\partial y}},v = - \frac{\partial \psi }{{\partial x}} $$

Following transformations has used to flow equations

14 $$ \begin{gathered} \psi \left( {x,y} \right) = \left( {\frac{4U\nu x}{3}} \right)^{\frac{1}{2}} F(\eta ),\,\,\,\,\,u = UF^{\prime}(\eta ){,}\,\,\,\,\,v = - \frac{1}{2}\left( {\frac{4U\nu }{{3x}}} \right)^{\frac{1}{2}} \left( {F(\eta ) - \eta F^{\prime}(\eta )} \right),\, \hfill \\ \eta = \left( {\frac{3U}{{4\nu x}}} \right)^{\frac{1}{2}} y,\,\,\,\,\theta (\eta ) = \frac{{T - T_{\infty } }}{{T_{w} - T_{\infty } }},\,\,\,\,\varphi (\eta ) = \frac{a}{a\infty },\,\,\,\,X(\eta ) = \frac{b}{a\infty },\,\,\,\,H(\eta ) = \frac{C - C\infty }{{\Delta C}}. \hfill \\ \end{gathered} $$

Incorporating Eq. (14) into our model we have the following set of ODEs

15 $$ \begin{gathered} F^{\prime\prime\prime}(\eta ) + 2/3\left( {1 - \phi_{1} } \right)^{2.5} \left( {1 - \phi_{2} } \right)^{2.5} \left[ {\left( {1 - \phi_{2} } \right)\left\{ {1 - \left( {1 - \frac{{\rho_{{MW{\text{C}}NT}} }}{{\rho_{f} }}} \right)\phi_{1} + \phi_{2} \frac{{\rho_{{SW{\text{C}}NT}} }}{{\rho_{f} }}} \right\}} \right] \hfill \\ \left[ {1 - \left( {F^{\prime}(\eta )} \right)^{2} + F(\eta )F^{\prime\prime}(\eta ) - 2\lambda (\eta )\theta (\eta )} \right] - 4/3\left( {1 - \phi_{1} } \right)^{2.5} \left( {1 - \phi_{2} } \right)^{2.5} MF^{\prime}(\eta ) - 3/4KF^{v} (\eta ) = 0, \hfill \\ \end{gathered} $$

16 $$ \theta^{\prime\prime}(\eta ) + 2/3\left[ {\left( {1 - \phi_{2} } \right)\left\{ {1 - \left( {1 - \frac{{\rho_{{MW{\text{C}}NT}} }}{{\left( {\rho c_{p} } \right)_{f} }}} \right)\phi_{1} + \phi_{2} \frac{{\rho_{{SW{\text{C}}NT}} }}{{\left( {\rho c_{p} } \right)_{f} }}} \right\}} \right]\Pr F(\eta )\theta^{\prime}(\eta ) + \Pr R_{H} \Phi (\eta )\left( {1 - \Phi (\eta )} \right)^{2} = 0, $$

17 $$ (1 - \phi 1)(1 - \phi 2)\Phi^{\prime\prime}(\eta ) + Sc\left[ {F(\eta )\Phi^{\prime}(\eta ) - K\Phi (\eta )\left( {1 - \Phi (\eta )} \right)^{2} } \right] = 0, $$

18 $$ H^{\prime\prime}(\eta ) + 2/3Lef(\eta )H^{\prime}(\eta ) = 0. $$

The transformed conditions are as fallow:

19 $$ \begin{gathered} F(\eta ) = 0,F^{\prime}(\eta ) = 0,\theta^{\prime}(\eta ) = - K_{T} \Phi (\eta ),\Phi^{\prime}(\eta ) = K_{s} \Phi (\eta ),H(\eta ) = 1,\,\,\,{\text{at}}\,\,\eta = 0, \hfill \\ F^{\prime}(\eta ) = 1,\theta (\eta ) = 0,\Phi (\eta ) = 1,H(\eta ) = 0,\,\,\,\,{\text{at}}\,\,\eta \to \infty . \hfill \\ \end{gathered} $$

In above process we have encountered some emerging parameters which are described in Table 1.

**Table 1:** Description of parameters.

Symbolic representation	Mathematical representation	Physical interpretation
$M$	$\sigma_{f} B_{0}^{2} x/\rho_{f} U$	Revised magnetic parameter
$K$	$\eta_{0} A_{1} U/x\rho_{f}$	Coefficient of homogeneous reaction
$\lambda$	$\frac{1}{2}\beta \Delta T$	Buoyancy parameter
$\Pr$	$v/a$	Prandtl number
$R_{H}$	$\frac{{2^{3/2} x^{1/2} a_{\infty }^{3} k_{1} \Delta H_{h} }}{{3g^{1/2} \rho_{f} c_{p} \delta_{A} \Delta T}}$	Homogeneous reaction heat parameter
$K_{T}$	$\frac{{2^{3/4} v^{1/2} x^{1/4} a_{\infty } k_{s} \Delta H_{h} }}{{3^{1/2} g^{1/4} \delta_{A} \Delta Tk_{T} }}$	Thermal conductivity
$Sc$	$v/D_{1}$	Schmidt number
$Le\,$	$\,v/D_{B}$	Lewis number
$K_{s}$	$\frac{{2^{3/4} v^{1/2} x^{1/4} k_{s} }}{{3^{1/2} g^{1/4} D_{1} }}$	Heterogeneous reaction parameter

It is to noticed that for holding similarity solutions we must have that $k_{1}$ and $x^{ - 1/2}$ are proportional to each other whereas $k_{s}$ and $x^{ - 1/4}$ are proportional to each other.

Thermophysical properties of the hybrid nanofluids are described as follows^38^

$$ \begin{gathered} \upsilon_{hnf} = \frac{{\mu_{hnf} }}{{\rho_{hnf} }},\,\,\,\,\,\,\,\,\,\,\,\,\,\,\mu_{hnf} = \frac{{\mu_{f} }}{{(1 - \phi_{1} )^{5/2} (1 - \phi_{2} )^{5/2} }},\,\,\,\,\,\,\,\frac{{(\rho )_{hnf} }}{{(\rho )_{f} }} = (1 - \phi_{2} )\left\{ {1 - \left( {1 - \frac{{(\rho )_{MWCNT} }}{{(\rho )_{f} }}} \right)\phi_{1} } \right\} + \frac{{(\rho )_{SWCNT} }}{{(\rho )_{f} }}\phi_{2} , \hfill \\ \hfill \\ \frac{{(\rho C_{p} )_{hnf} }}{{(\rho C_{p} )_{f} }} = (1 - \phi_{2} )\left\{ {1 - \left( {1 - \frac{{(\rho C_{p} )_{MWCNT} }}{{(\rho C_{p} )_{f} }}} \right)\phi_{1} } \right\} + \frac{{(\rho C_{p} )_{SWCNT} }}{{(\rho C_{p} )_{f} }}\phi_{2} , \hfill \\ \hfill \\ \frac{{k_{hnf} }}{{k_{bf} }} = \frac{{(1 - \phi_{2} ) + 2\phi_{2} \frac{{k_{SWCNT} }}{{(k_{SWCNT} - k_{bf} )}} - \ln \frac{{k_{SWCNT} + k_{bf} }}{{2k_{{_{bf} }} }}}}{{(1 - \phi_{2} ) + 2\phi_{2} \frac{{k_{bf} }}{{(k_{SWCNT} - k_{bf} )}} - \ln \frac{{k_{SWCNT} + k_{bf} }}{{2k_{{_{bf} }} }}}},\,\,\,\,\,\,\,\,\,\,\,\,\frac{{k_{bf} }}{{k_{f} }} = \frac{{(1 - \phi_{1} ) + 2\phi_{1} \frac{{k_{MWCNT} }}{{(k_{SWCNT} - k_{f} )}} - \ln \frac{{k_{MWCNT} + k_{f} }}{{2k_{{_{bf} }} }}}}{{(1 - \phi_{1} ) + 2\phi_{1} \frac{{k_{bf} }}{{(k_{MWCNT} - k_{f} )}} - \ln \frac{{k_{MWCNT} + k_{f} }}{{2k_{{_{f} }} }}}}, \hfill \\ \end{gathered} $$

In above equations $k_{hnf} ,\,\,k_{f}$ are thermal conductivities for $Fe_{3} O_{4}$ and $H_{2} O$. Also $\phi_{1}$ and $\phi_{2}$ are the respective volume fractions for $Fe_{3} O_{4}$ and CNTs. The thermophysical properties for pure fluid and CNTs are presented in Table 2.

**Table 2:** The numerical properties of blood and $CNTs$.

Properties	Pure fluid	Hybrid nanofluid	
	Blood	MWCNTs	SWCNTs
$\rho \;(kg/m^{3} )$	1050	1600	2600
$k\;(W/mK)$	0.52	3000	6600
$C_{p} \;(j/kgK)$	3617	796	425

### Required physical quantities

The skin-friction coefficient $C_{fx}$ and local Nusselt number $Nu_{x}$, Sherwood number $Sh_{x}$ are given mathematically as:

21 $$ C_{fx} = \frac{{\tau_{w} }}{{\rho_{hnf} \left( U \right)^{2} }}, \; Nu_{x} = \frac{{xq_{w} }}{{k_{hnf} \left( {T_{w} - T_{\infty } } \right)}}, \; Sh_{x} = \frac{{xj_{w} }}{{D_{B} \left( {C_{w} - C_{\infty } } \right)}} $$

In Eq. (21) $\tau_{w}$ and $q_{w}$ reveal shear stress and heat flux respectively. Making use of Eq. (14) and incorporating values of $\tau_{w}$ and $q_{w}$ in Eq. (21) we finally have

22 $$ C_{fx} Re_{x}^{0.5} = \frac{\sqrt 3 }{{2\left( {1 - \phi_{1} } \right)^{2.5} \left( {1 - \phi_{2} } \right)^{2.5} }}F^{\prime\prime}\left( 0 \right),\,\,\,Nu_{x} Re_{x}^{ - 0.5} = - \frac{{k_{hnf} }}{{k_{f} }}\frac{\sqrt 3 }{2}\theta^{\prime}\left( 0 \right),\,\,\,Sh_{x} Re_{x}^{ - 0.5} = - \frac{\sqrt 3 }{2}\Phi^{\prime}\left( 0 \right). $$

### Method for solution

In this work the modeled equations are converted to dimensionless notation by using some useful dimensionless variables as given in Eq. (14). After this transformation we have obtained Eqs. (15–19). These Eqs. (15–18) are then solved by semi analytical technique HAM ^39,40^ by employing the boundary conditions as given in Eq. (19). Following initial guesses have sued:

23 $$ f_{0} \left( \eta \right) = 1 - e^{\eta } ,\,\,\,\Theta_{0} \left( \eta \right) = \frac{{\pi_{1} }}{{1 + \pi_{1} }}e^{ - \eta } ,\,\,\,\Phi_{0} \left( \eta \right) = \frac{{\pi_{2} }}{{1 + \pi_{2} }}e^{ - \eta } $$

Here

24 $$ L_{f} \left( f \right) = f^{\prime\prime\prime} - f^{\prime},\,\,L_{\Theta } \left( \Theta \right) = \theta^{\prime\prime} - \theta ,\,\,L_{\Phi } \left( \Phi \right) = \phi^{\prime\prime} - \phi $$

The expanded forms of these operators as stated in Eq. (24) are expressed as follows

25 $$ L_{f} \left( {c_{1} + c_{2} e^{\eta } + c_{3} e^{ - \eta } } \right) = 0,\,\,L_{\Theta } \left( {c_{4} e^{\eta } + c_{5} e^{ - \eta } } \right) =0 ,\,\,\,L_{\Phi } \left( {c_{6} e^{\eta } + c_{7} e^{ - \eta } } \right) = 0 $$

Above in Eq. (25) $c_{i} \,\,for\,\,i = 1,\,2,\,3, \ldots 7$ are constants.

By Taylor series expansion we have

26 $$ \begin{gathered} f(\eta ;\xi ) \, = \, f_{0} (\eta ) + \sum\nolimits_{n = 1}^{\infty } {f_{n} (\eta )\xi^{n} } \hfill \\ \Theta (\eta ;\xi ) \, = \, \Theta_{0} (\eta ) + \sum\nolimits_{n = 1}^{\infty } {\Theta_{n} (\eta )\xi^{n} } \hfill \\ \Phi (\eta ;\xi ) \, = \, \Phi_{0} (\eta ) + \sum\nolimits_{n = 1}^{\infty } {\Phi_{n} (\eta )\xi^{n} } \hfill \\ \end{gathered} $$

## Results and discussion

This study determines the MHD flow of gravity-driven couple stress hybrid nanofluid past a heated plate. Carbon nanotubes (CNTs) are used to characterize the hybrid nanofluid. The heated plate is placed vertically with an application of homogenous-heterogeneous reactions to the assumed flow system. An appropriate set of dimensionless variables has employed to governing equations in order to achieve a set of dimensionless ODEs and its solution has then carried out by HAM. The effects of emerging parameters encountered in this work are discussed in detail with the help of graphs.

### Flow profile $F^{\prime}\left( \eta \right)$

**Figure 2:**
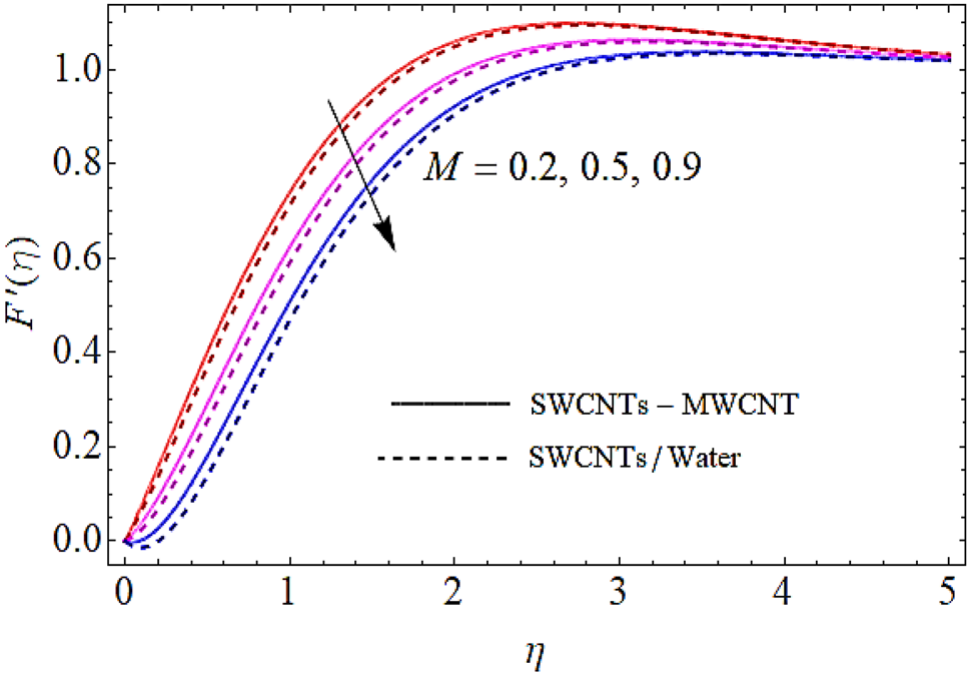
Flow characteristics versus $M$ when $\lambda = 0.2,\,\,\,\varphi_{1} = 0.02,\,\,\varphi_{2} = 0.01,\,\,K = 0.7$.

**Figure 3:**
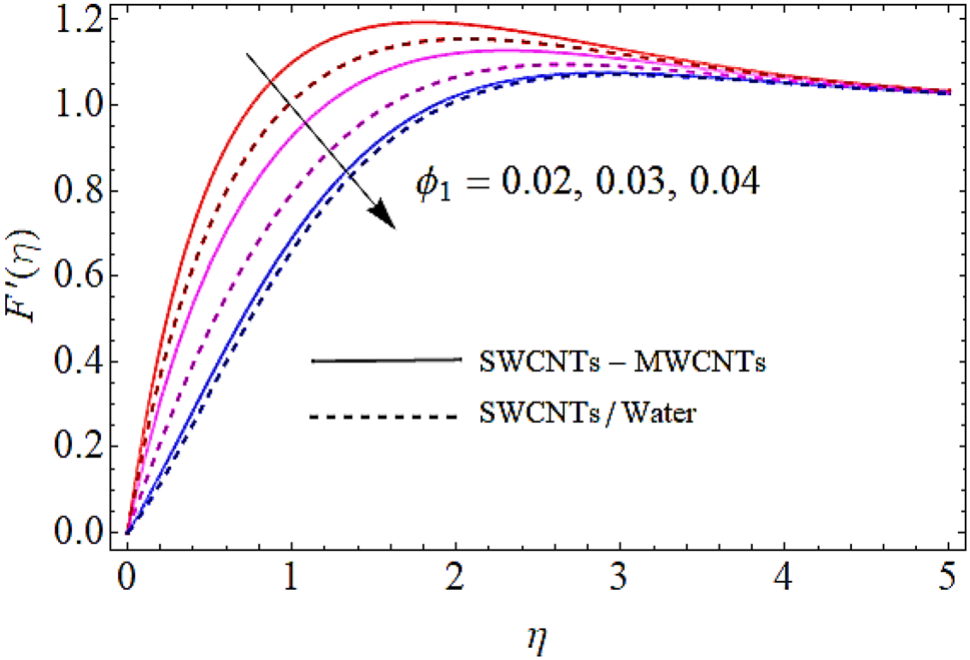
Flow characteristics versus $\phi_{1}$ when $\lambda = 0.2,\,\,\,M = 0.3,\,\,\varphi_{2} = 0.01,\,\,K = 0.7$.

**Figure 4:**
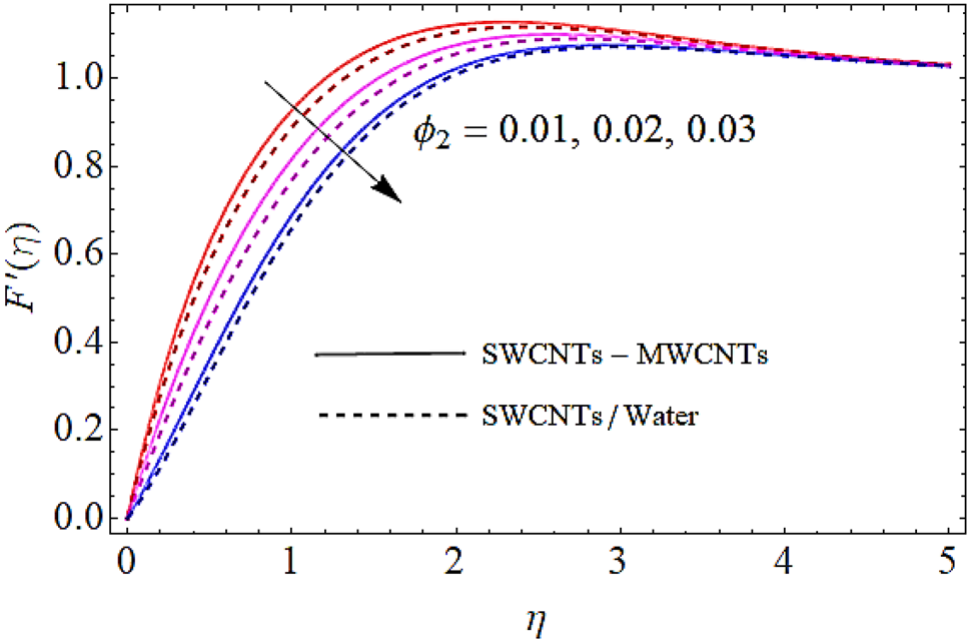
Flow characteristics versus $\phi_{2}$ when $\lambda = 0.2,\,\,\,\varphi_{1} = 0.02,\,\,M = 0.3,\,\,K = 0.7$.

**Figure 5:**
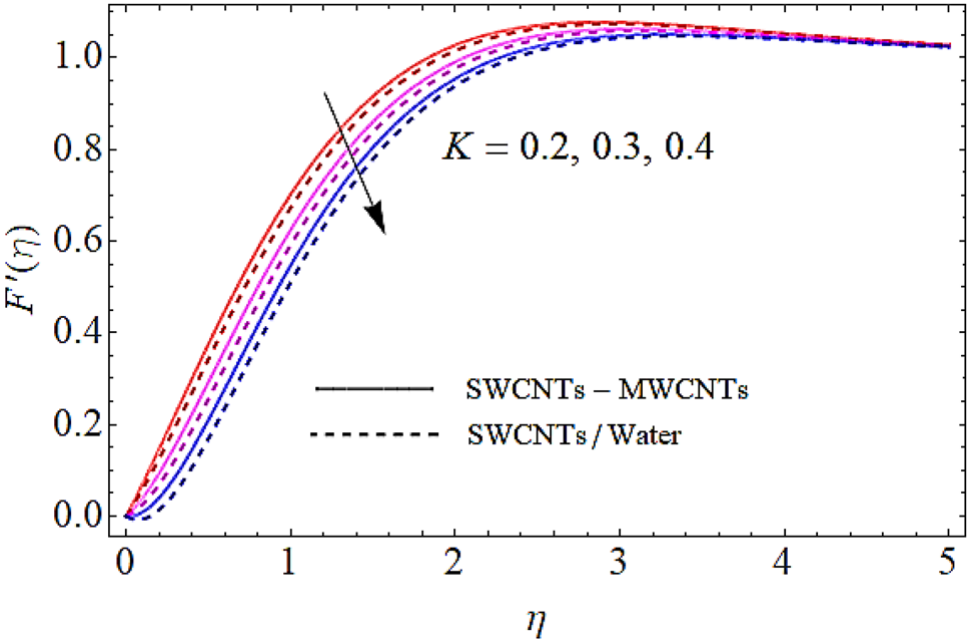
Flow characteristics versus $K$ when $\lambda = 0.2,\,\,\,\varphi_{1} = 0.02,\,\,\varphi_{2} = 0.01,\,\,M = 0.3$.

In Figs. 2, 3, 4, 5 we shall discuss the effects of magnetic field $\left( M \right)$, volume fractions $\left( {\phi_{1} ,\,\,\phi_{2} } \right)$ and coefficient of homogeneous reaction parameter $\left( K \right)$ on flow of fluid. Since the augmentation in magnetic effects give rise to the production of Lorentz force in fluid flow that declines the fluid motion. Hence increase in $\left( M \right)$ results in drop of flow as determines in Fig. 2. This impact is more visible for MWCNTs than SWCNTs, because SWCNTs are denser than MWCNTs. It is also to be noticed that with augmentation in volumetric concentrations of $Fe_{3} O_{4}$ and CNTs denoted by $\phi_{1} ,\,\,\phi_{2}$ respectively, the viscosity of the fluid is augmented. With increase in viscous forces, the fluid motion also reduces both for SWCNTs and MWCNTs as depicted in Figs. 3, 4. Figure 5 represents the impact of coefficient of homogeneous reaction parameter $\left( K \right)$ upon flow characteristics. It is revealed that increase in $\left( K \right)$ results a reduction in velocity profile both for SWCNTs and MWCNTs.

### Thermal profile $\theta \left( \eta \right)$

**Figure 6:**
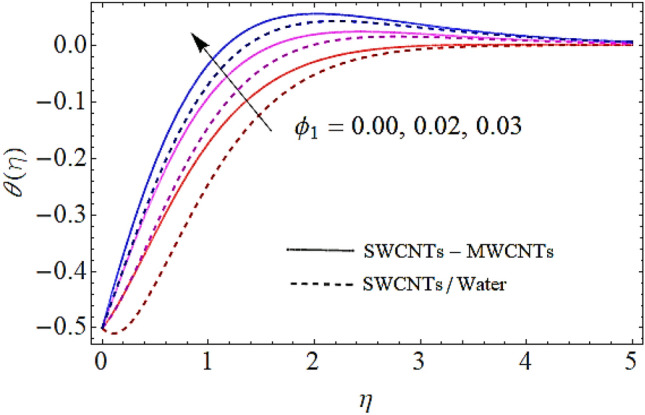
Thermal profiles versus $\phi_{1}$ when $M = 0.5,\,\lambda = \,0.3\,,\phi_{2} = \,0.02\,,K = 0.7,\Pr = \,12\,,R_{H} = 0.6$.

**Figure 7:**
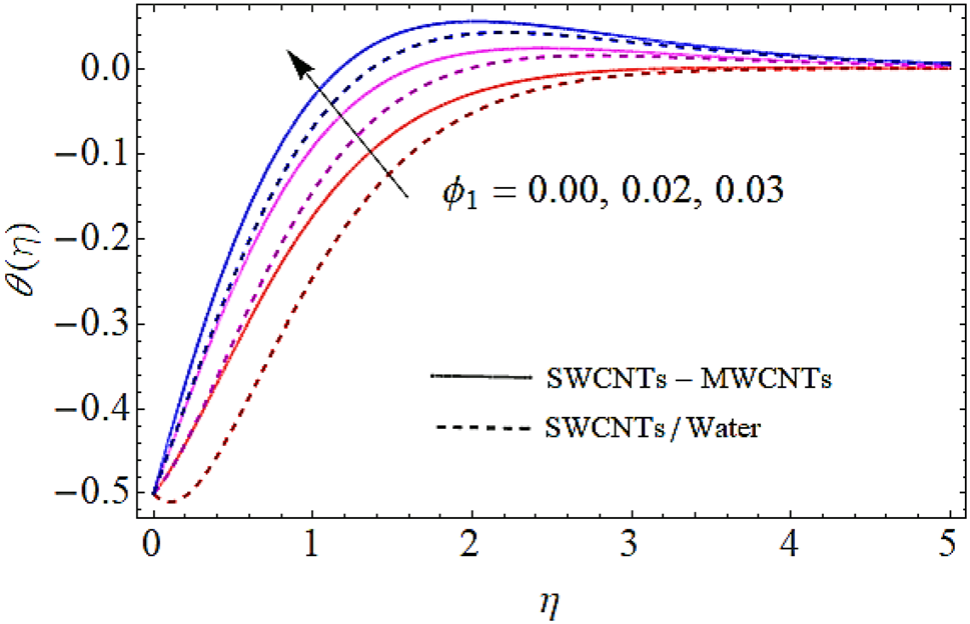
Thermal profiles versus $\phi_{2}$ when $M = 0.5,\,\lambda = \,0.3\,,\,\phi_{1} = \,0.02\,,K = 0.7,\Pr = \,12\,,R_{H} = 0.6$.

**Figure 8:**
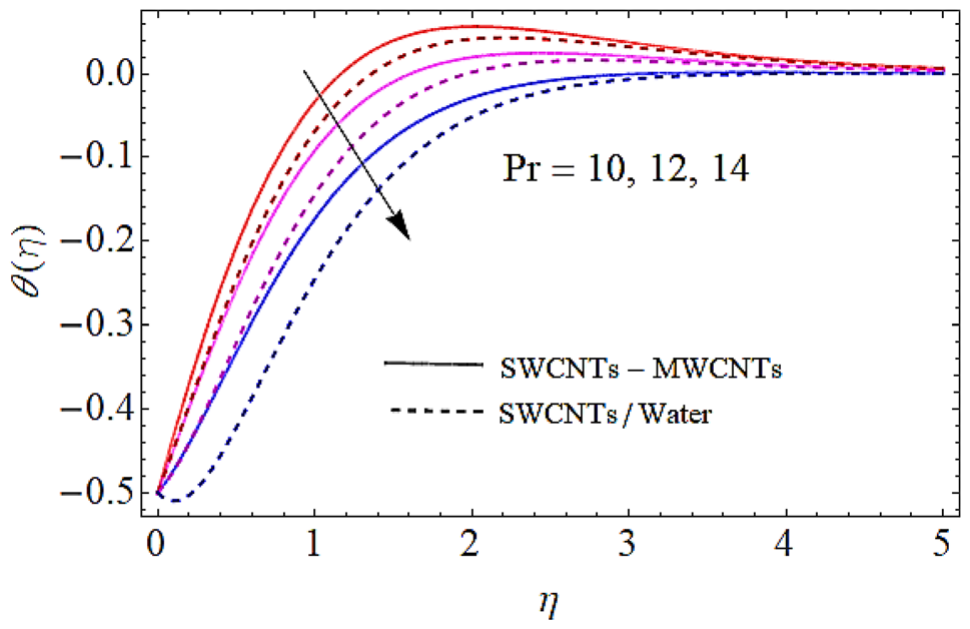
Thermal profiles versus $\Pr$ when $M = 0.5,\;\lambda = \,0.3\,,\,\;\phi_{1} = \phi_{2} = \,0.02\,,K = 0.7,R_{H} = 0.6$.

**Figure 9:**
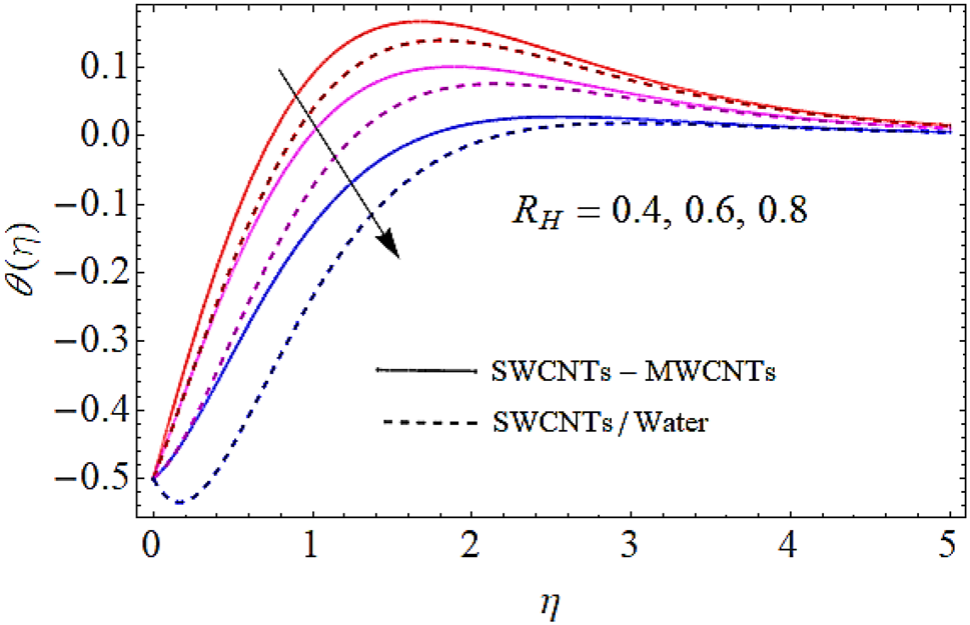
Thermal profiles versus $R_{H}$ when $M = 0.5,\,\lambda = \,0.3\,,\,\phi_{1} = \phi_{2} = \,0.02\,,K = 0.7,Pr = 12$.

Next we shall discuss the effects of volume fractions $\left( {\phi_{1} ,\,\,\phi_{2} } \right)$, Prandtl number $\left( {\Pr } \right)$ and homogeneous reaction heat parameter $\left( {R_{H} } \right)$ upon thermal profile as expressed in Figs. 6, 7, 8, 9. The density of SWCNTs and MWCNTs grow up with corresponding augmentation in volumetric concentration of ${\text{Fe}}_{3} {\text{O}}_{4}$ and CNTs denoted by $\phi_{1} ,\,\,\phi_{2}$ respectively. Hence with growth in volume fractions of nanofluid there is an augmentation in temperature of the fluid as depicted in Figs. 6, 7. The growing values of Prandtl number declines the thermal diffusivity and mass of the nanofluid. So growth in $\Pr$ reduces the thermal flow as shown in Fig. 8. It is also to be noticed that homogeneous reaction heat parameter is inversely proportional to the difference of heat transfer. Hence augmentation in $\left( {R_{H} } \right)$ leads to a decline in heat transfer as depicted in Fig. 9.

### Concentration profile $\varphi \left( \eta \right)$

**Figure 10:**
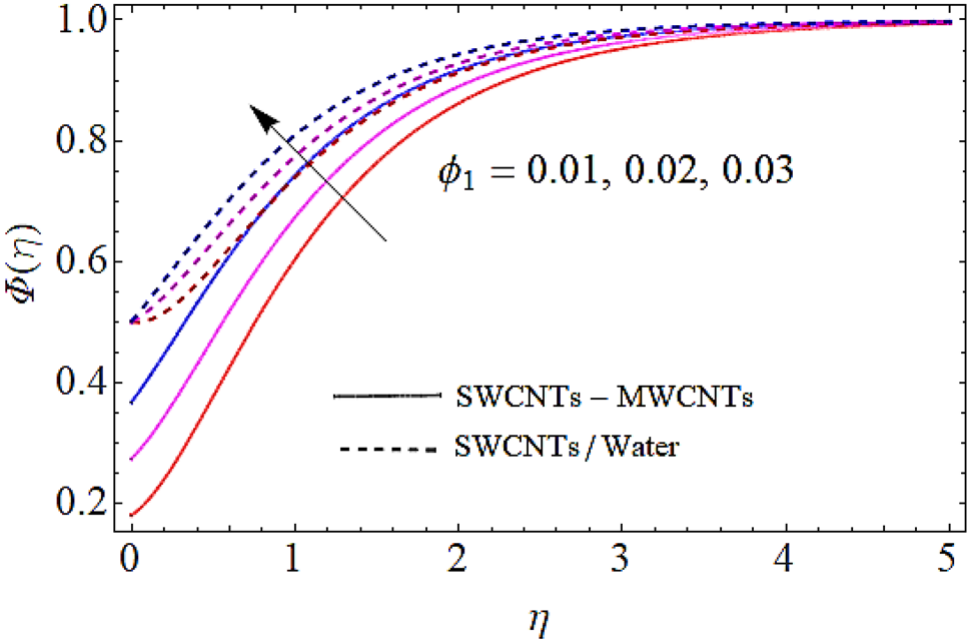
Concentration profile versus $\phi_{1}$ when $M = 0.5,\,\phi_{2} = \,0.02\,,K = 0.7,Sc = 0.7,Pr = 12$.

**Figure 11:**
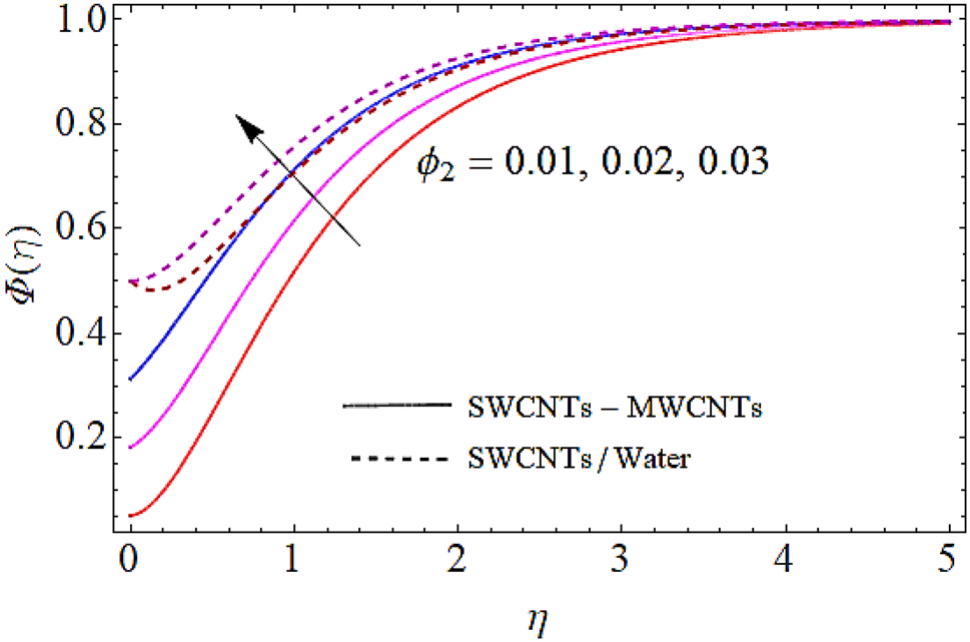
Concentration profile versus $\phi_{2}$ when $M = 0.5,\,\phi_{1} = \,0.02\,,K = 0.7,Sc = 0.7,Pr = 12$.

**Figure 12:**
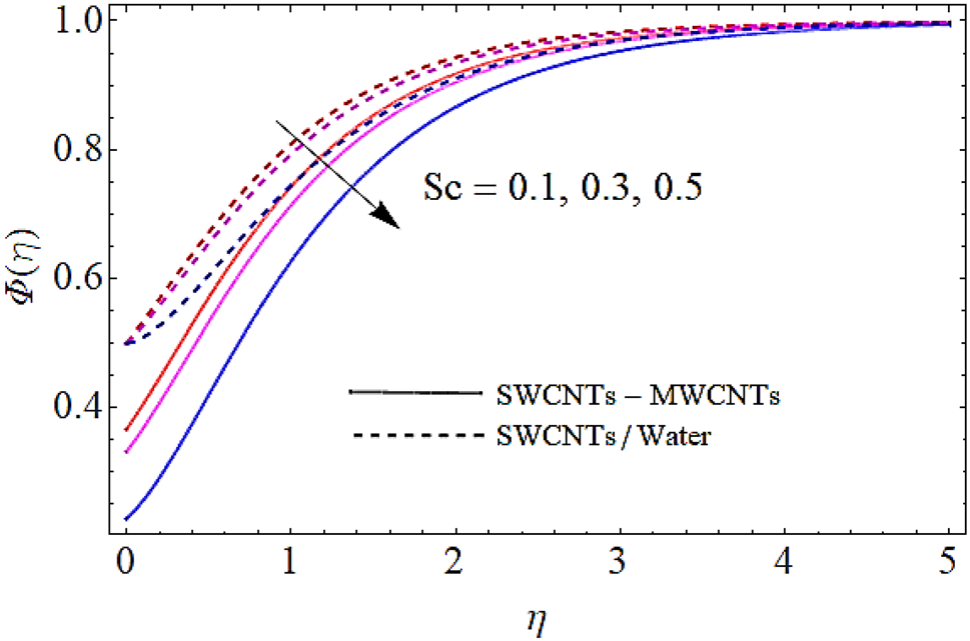
Concentration profile versus $Sc$ when $M = 0.5,\,\phi_{1} = \phi_{2} = \,0.02\,,K = 0.7,Pr = 12$.

**Figure 13:**
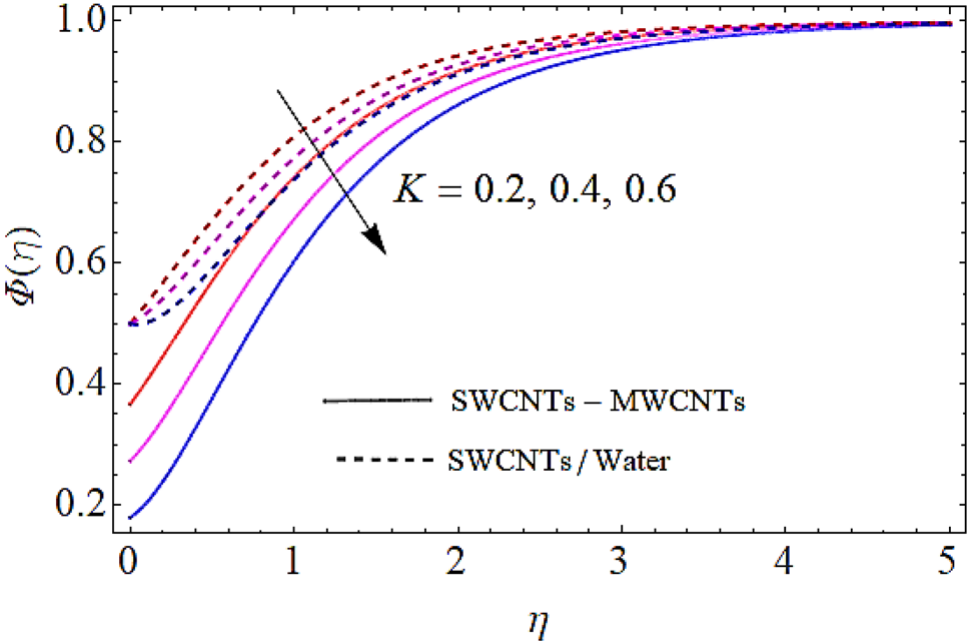
Concentration profile versus $K$ when $M = 0.5,\,\phi_{1} = \phi_{2} = \,0.02\,,Sc = 0.7,Pr = 12$.

Next we shall discuss the influence of volume fractions $\left( {\phi_{1} ,\,\,\phi_{2} } \right)$, Schmidt number $\left( {Sc} \right)$ and coefficient of homogeneous reaction $\left( K \right)$ upon concentration profile as given in Figs. 10, 11, 12, 13. The density of SWCNTs and MWCNTs grow up with corresponding augmentation in volumetric concentration of ${\text{Fe}}_{3} {\text{O}}_{4}$ and CNTs denoted by $\phi_{1} ,\,\,\phi_{2}$ respectively. In this process the concentration boundary layer of hybrid nanofluid also grows up. Hence with augmentation in volume fraction of nanofluid, there is an augmentation in concentration of the fluid as depicted in Figs. 10, 11. It is to be noticed that with augmentation in Schmidt number the mass diffusivity of the liquid reduces. Hence growth in the Schmidt number results a reduction in concentration profile as depicted in Fig. 12. Moreover, the augmentation in coefficient of homogeneous reaction parameter leads to a drop down in concentration as depicted in Fig. 13.

### Discussion of tables

Table 3 portrays numerically, the impact of different values of emerging parameters upon coefficient of skin friction. From this table it is revealed that with augmentation in values of buoyancy parameter there is a growth in the values of skin friction coefficient. From Table 4 it is observed that with increment in Prandtl number the thermal boundary layer decreases due to which Nusselt number reduces gradually. Moreover, Table 5 portrays that Sherwood number reduces numerically with a corresponding augmentation in Schmidt number because the concentration boundary layer gets thinner with augmentation in Schmidt number. The comparison between the results of current investigation and those published in the literature^41,42^ has been carried out in Table 6. A reasonable promise among these results has been noticed in this work.

**Table 3:** Impact upon skin friction regarding different values of emerging parameters.

$\lambda$	$\varphi_{1} + \varphi_{2}$	$M$	$K$	$1/2{\text{Re}}_{x}^{1/2} C_{{f_{x} }}$ for $SWCNTs$	$1/2{\text{Re}}_{x}^{1/2} C_{{f_{x} }}$ for $SWCNTs + MWCNTs$
0.0	0.01	0.1	0.1	0.9988	1.2346
0.5				1.0325	2.3457
0.9				1.2436	2.8578
	0.02			1.3547	2.9689
	0.03			1.4758	3.1799
	0.05			1.5869	3.2831
		0.2		1.6972	3.3943
		0.3		1.8281	3.4452
		0.4		1.9321	3.5661
			0.2	2.0236	3.6782
			0.3	2.1345	3.7971
			0.4	2.2435	3.8182

**Table 4:** Impact upon Nusselt number regarding different values of emerging parameters.

$\Pr$	$\varphi_{1} + \varphi_{2}$	$Nu_{x} {\text{Re}}_{x}^{ - 1/2}$ for $SWCNTs$	$Nu_{x} {\text{Re}}_{x}^{ - 1/2}$ for $SWCNTs + MWCNTs$
6.5		0.5824	0.4632
6.7		0.4735	0.3521
6.8		0.3642	0.2431
6.9	0.02	0.4721	0.4342
	0.03	0.4832	0.4931
	0.04	0.5941	0.6021

**Table 5:** Impact upon Sherwood number regarding different values of emerging parameters.

$Sc$	$\varphi_{1} + \varphi_{2}$	$Sh_{x} {\text{Re}}_{x}^{ - 1/2}$ for $SWCNTs$	$Sh_{x} {\text{Re}}_{x}^{ - 1/2}$ for $SWCNTs + MWCNTs$
0.1	0.01	0.7215	0.6322
0.2		0.6126	0.5211
0.3		0.5237	0.4123
	0.02	0.4348	0.3214
	0.03	0.3459	0.2325

**Table 6:** Comparison of the ($1/2{\text{Re}}_{x}^{1/2} C_{{f_{x} }}$) for present work with published work. When $\Pr = 10.2,\;Nt = Nb = 0.2,\;RH = Le = 0.5,\;Ks = 0.045$.

$\lambda$	Raees and Hang^41^	Sohail and Hang^42^	Present
0.0	0.9887653	0.9887964	0.98879988212
0.3	1.0143357	1.01433875	1.0143431240
0.5	1.0321132	1.03212431	1.0321314210

## Conclusion

An analytical study is carried out in current article for a gravity driven MHD flow of a coupled stress CNTs over a heated plate that is placed vertically upward. The homogenous-heterogeneous reactions are also assumed for current flow system. The modeled equations are changed to set of ODEs with the help of dimensionless quantities and then have determined its solution by employing HAM. The effects of emerging parameters encountered in the work are discussed in detail with the help of graphs. Following points are noticed after detail study of the work.

- Since the augmentation in magnetic effects leads to production of Lorentz force in fluid flow that opposes the fluid motion. Hence growth in magnetic parameter drops down the flow both for SWCNTs and MWCNTs.
- It is also to be noticed in this work that, with augmentation in volumetric concentrations the viscosity of the fluid is augmented and hence the fluid motion reduces.
- The augmentation in homogeneous reaction leads to decline in velocity profile both for SWCNTs and MWCNTs.
- The density of SWCNTs and MWCNTs grow up with corresponding augmentation in volumetric concentration that enhances thermal boundary layer and hence grows thermal characteristics.
- The augmentation in Prandtl number and homogeneous reaction heat parameter corresponds to a reduction in thermal characteristics of hybrid nanofluid. Moreover, enhancement in Prandtl number also corresponds to decline in Nusselt number.
- The density of SWCNTs and MWCNTs grow up with corresponding augmentation in volumetric concentration of CNTs that leads to growth in mass transfer.
- It is to be noticed that with augmenting values of Schmidt number the mass diffusivity of the liquid reduces and causes a decline in concentration boundary layer. Hence growth in Schmidt number leads to drop down in concentration as well as in Sherwood number, both for SWCNTs and MWCNTs.
- The impact of various physical parameters upon quantities of interest has been calculated numerically in the tabular form.
- In future, the influence of variable thermal conductivity, induced magnetic field and Hall effects can be incorporated for extension of current study. Moreover, the effects of Casson fluid can also be included in the current work.

## Data Availability

The data that support the findings of this investigation is available from the corresponding author upon reasonable request.
